# Hip Fracture Leads to Transitory Immune Imprint in Older Patients

**DOI:** 10.3389/fimmu.2020.571759

**Published:** 2020-09-18

**Authors:** Héléne Vallet, Charles Bayard, Héléne Lepetitcorps, Jessica O'Hana, Soléne Fastenackels, Tinhinane Fali, Judith Cohen-Bittan, Frédéric Khiami, Jacques Boddaert, Delphine Sauce

**Affiliations:** ^1^Sorbonne Université, INSERM, Centre d'Immunologie et des Maladies Infectieuses (Cimi-Paris), Paris, France; ^2^Assistance Publique Hôpitaux de Paris (APHP), Hôpital Saint Antoine, Department of Geriatrics, Paris, France; ^3^Assistance Publique Hôpitaux de Paris (APHP), Hôpital Pitié-Salpétrière, Department of Geriatrics, Paris, France; ^4^APHP, Hôpital Pitié-Salpétrière, Department of Orthopedic Surgery, Paris, France

**Keywords:** acute stress, immune response, aging, inflammation, regulation loop

## Abstract

**Background:** Hip fracture (HF) is common in the geriatric population and is associated with a poor vital and functional prognosis which could be impacted by immunological changes. The objective here is to decipher immune changes occurring in the 1st days following HF and determine how phenotype, function, and regulation of innate and adaptive compartments adapt during acute stress event.

**Methods:** We included HF patients, aged over 75 years. For each patient, blood samples were taken at five different timepoints: four in the perioperative period (day 0 to hospital discharge) and one at long term (6–12 months). Phenotypical and functional analysis were performed longitudinally on fresh blood or cryopreserved PBMCs. Clinical data were prospectively collected.

**Results:** One-hundred HF patients and 60 age-matched controls were included. Innate compartment exhibits pro-inflammatory phenotypes (hyperleukocytosis, increase of CD14+ CD16+ proportion and CCR2 expression), maintaining its ability to produce pro-inflammatory cytokines. Adaptive compartment extends toward a transitory immunosuppressive profile (leucopenia) associated with an active T-cell proliferation. Furthermore, increases of LAG-3 and PD-1 and a decrease of 2-B4 expression are observed on T-cells, reinforcing their transitory suppressive status. Of note, these immune changes are transitory and sequential but may participate to a regulation loop necessary for homeostatic immune control at long term.

**Conclusion:** HF is associated with several transitory immunological changes including pro-inflammatory phenotype in innate compartment and immunosuppressive profile in adaptive compartment. A comprehensive assessment of immune mechanisms implicated in the patient's prognosis after HF could pave the way to develop new immune therapeutics strategies.

## Introduction

Worldwide, 1.6 million of patients suffer of hip fracture (HF) each year, notably in the aged population (1). This frequent pathology is associated with a poor prognosis with high mortality rate (20–30% of one-year mortality) and a decrease of functional autonomy (2–4). Main factors associated with death are not directly due to the HF and/or its treatment but are represented by comorbidities decompensations (cardio-vascular events) and secondary infections (5). The fall, and consequently the HF generates an important acute stress that impacts organism and could induce immunological changes in this context (6, 7).

Immunosenescence, defined as the impact of age on the immune system, is characterized by phenotypical and functional changes that affect innate and adaptive compartments. Briefly, phagocytosis and chimiotactism of innate cells (monocytes, macrophages, or neutrophils) are decreased. The pool of naïve T cells decreases due to the thymic involution (8) and there is a shrinking of TCR repertoire (9). Furthermore, older patients present an elevated level of pro-inflammatory cytokines coined “Inflam-aging” (10).

Several studies have shown immunological changes after HF. Neutrophils exhibit functional alterations with a defect in phagocytosis ability and superoxide production (6). Conventional monocytes switch toward inflammatory phenotype with an increased production of tumor necrosis factor alpha (TNF-α) (11).

Finally, HF prognosis has been associated with an increase of pro-inflammatory cytokines (IL-6, TNF-α) and few biomarkers have been described (c-reactive protein, procalcitonin) (7, 12). In a previous study, we observed that pre-operative rate of neopterin (a molecule secreted by myeloid lineage under IFN-γ stimulation) was strongly associated with long-term mortality (13). The objective of this longitudinal study is to decipher immune changes occurring in the 1st days following HF and determine how phenotype, function, and regulation of innate and adaptive compartments adapt during acute stress event.

## Materials and Methods

### Patients Cohort

We included hip fracture patients, aged over 75 years admitted between 2013–2015 and 2017–2018 in emergency department of Pitié Salpêtrière hospital. Patients with metastatic fracture, history of cancer, autoimmune disease, and/or immunosuppressive treatment were excluded. For each patient, five blood sample were taken at different timepoints: in pre-operative period (Pre), 24 h after surgery (Post), between day 3 and 5 of hospitalization usually (Hosp), then at patient hospital discharge (Discharge) and finally at long term post-surgery (6–12 months; Long term). Healthy individuals matched for age were included in geriatric department and a unique blood sample was collected. One milliliter of fresh blood was immediately used for cell count and innate phenotyping. PBMCs (isolated by density gradient centrifugation) and plasma were cryopreserved until use.

Clinical data including age, sex, comorbidity scale (Cumulative Illness Rating Scale, CIRS), functional status and frailty scale {Activity of daily living [ADL (14)], Instrumental activity of daily living [IADL (15)], Clinical Frailty Scale (CFS) (16)^1^} at admission and functional and vital status at long term (6–12 months) were prospectively collected.

This study was approved by the ethics committee (CPP Pitié-Salpêtrière, Paris, France). All participants included were informed and gave their consent. The database was recorded to the French National Commission for Computing and Liberty (CNIL, Paris, France).

### Flow Cytometry Analysis

#### Staining on Fresh Cells

The percentages and absolute counts of lymphocyte subpopulations were determined in whole blood using CytoStat tetra-CHROME reagents (panel 1: CD45-FITC/CD56-PE/CD19-ECD and CD3-PC5; panel 2: CD45-FITC/CD4-RD1/CD8-ECD and CD3/PC5; Beckman Coulter, Hialeah, Florida). Sample acquisition with Flow-Count Fluorospheres was performed on a FC500 flow cytometer (Beckman Coulter).

#### Cell Counts

##### Innate phenotyping

Directly conjugated antibodies were obtained from the following vendors: BD Biosciences (San Jose, CA): CD3 (FITC), CD14 (BV605), HLADR (PE-CF594), CD16 (APC-H7); R&D systems: CCR2 (APC); eBioscience (San Diego, CA): CX3CR1 (PE), CD62L (PerCP-eF710). Staining for innate cell surface markers was performed with 100 μl of fresh blood incubated 15 min with antibodies. The blood was then lysed with BD FACS lysing, resuspended with PBS 1X (BD Biosciences) and immediately analyzed by flow cytometry.

#### Staining on Frozen Cells

##### Immune checkpoint phenotype

Directly conjugated antibodies were obtained from the following vendors: BD Biosciences (San Jose, CA): CCR7 (PC7), CD45-RA (V450), CD4 (HV500), HLA-DR (BV650), CD8 (APC-Cy7); BioLegend (San Diego, CA): PD1 (BV711), LAG3 (FITC), CD244/2B4 (PE). Staining was performed on cryopreserved PBMCs with standard method.

##### Homeostatic proliferation assay and activation status

Directly conjugated antibodies were obtained from the following vendors: BD Biosciences (San Jose, CA): CD4 (APC-cyanin7), CCR7 (PE-Cy7), CD38 (APC), and Ki67 (FITC); Beckman Coulter: CD45RA (ECD), Caltag (Burlingame, CA): CD8 (Alexa405); Dako (Glostrup, Denmark): CD3 (Cascade Yellow); BioLegend (San Diego, CA): CD27 (AlexaFluor700). Cell surface marker stainings were performed by addition of the respective antibodies for 15 min at room temperature. After incubation, cells were washed in PBS and then permeabilized with Perm/fix kit (eBiosciences) before the addition of Ki67 antibody. Stainings were analyzed on an LSR2 flow cytometer (Becton Dickinson) with appropriate isotype controls and color compensation.

##### CMV responsiveness

To assess functional capacity of HCMV specific CD8+ or CD4+ T cells, PBMC were stimulated with 15 amino acid long synthetic peptides (5 μM) overlapping by 10 amino acids and spanning the two HCMV proteins, pp65. After 1 h, the secretion inhibitor brefeldin A (5 μg/mL; Sigma-Aldrich) was added and the incubation was continued overnight at 37°C in a 5% CO_2_ atm. Cytofix/Cytoperm™ (BD Biosciences) was used to fix/permeabilize the cells prior to staining for intracellular IFN-γ and TNF-α. The limit of detection for cytokine secretion was 0.01% in CD8+ or CD4+ T cell populations. “FunkyCellsBoolean Dataminer” software (www.FunkyCells.com), provided by Dr. Martin Larsen (INSERM U1135, Paris, France), was used to determine the Polyfunctionality Index in response to pp65 stimulation (17).

##### Monocyte function

PBMCs (1 × 106/well) were stimulated overnight at 37°C with LPS 10μg/ml (*E. coli* serotype O55:B5; Alexis Biochemicals). After 1 h, 5μg/ml of brefeldin (Sigma-Aldrich) was added. After 12 hours of stimulation, cell surface staining was performed with CD14-BV605, HLA-DR BV650, CD16-APCH7, CD3-AF700 (BD Biosciences San Jose,CA). After washes in PBS, cells were fixed in Cytofix/Cytoperm buffer (BD Biosciences), for 30 min at 4°C, washed in PermWash buffer (BD Biosciences, and then stained for intra-cellular markers: IL8-BV510, TGFβ-PE-CF594, TNFα-APC (BD Biosciences San Jose, CA); IL10-AF488 (R&D systems); IL1β-PE (eBioscience, San Diego,CA); IL6-PECy7 (Biolegend, San Diego, CA) for 30 min at 4°C. After wash, PBMCs were resuspended in PBS, before their flow cytometry acquisition on LSR2 flow cytometer. Data were analyzed using FlowJo v9 (Tree Star, Inc.) and DIVA softwares (BD Biosciences).

### Statistical Analysis

Data are expressed as frequencies and percentages for categorical variables and as medians and interquartile ranges (IQR) for continuous variables. Bivariable associations were evaluated with the use of Mann–Whitney *U*-test for continuous variables. All analyses were performed at a two-sided alpha level of 5%. A *P*-value of <0.05 was considered to significant. All analyses were performed with SPSS software, version 20, and Graph Pad Prism, version 5.

## Results

### Patients Characteristics

One hundred patients with HF and 60 age-matched controls were included in this study. Main clinical characteristics are reported in Table 1. Patients with HF were 87 years old [83–92] and 27% were men. As expected, patients exhibit characteristics of geriatric population: they were comorbid (the median CIRS = 9 [7–13], polymedicated for 63% of them, and mildly frailed (a median CFS = 5 [4–6]. At 12-months, 19 of them were deceased. Patients and age-matched controls were comparable in terms of global comorbidities (CIRS) and functional autonomy (ADL and IADL) despite a higher clinical frailty score for HF.

**Table 1:** Patients characteristics.

**Variable**	**Hip fracture group (*n* = 110)**	**Control group (*n* = 60)**	***p-*value**
Age (years)	87 [83–92]	84 [80–89]	0.05
Male sex	30 (27.3%)	17 (28.3%)	0.9
**Comorbidities**			
CIRS	9 [7–13]	9 [6–12]	0.3
Dementia	46 (41.8%)	35 (58.3%)	0.02
Hypertension	74 (67.3%)	33 (55%)	0.1
Diabetes	16 (14.5%)	7 (11.7%)	0.6
Chronic cardiac failure	21 (19.1%)	5 (8.3%)	0.06
Chronic renal failure	67 (60.9%)	42 (70%)	0.2
COPD	8 (7.3%)	5 (8.3%)	0.8
Polymedication (≥5 drugs)	70 (63.6%)	37 (61.7%)	0.8
**Frailty/functional autonomy**			
CFS	5 [4–6]	4 [4–5]	0.03
ADL	6 [3.5–6]	6 [5–6]	0.2
IADL	2 [0.75–4]	3 [1–4]	0.3
Ability to walk	103 (93.6%)	59 (98.3%)	0.5
**Type of fracture**			
Intertrochanteric	55 (50%)		
Femoral neck	55 (50%)		
Number of post-operative complication	2 [1–4]		
**Long term outcomes**			
6-months mortality	17 (15.5%)		
12-months mortality	19 (17.3%)		
ADL at M6 (*n* = 48)	3 [2–6]		
IADL at M6 (*n* = 48)	1 [0–3.25]		
Ability to walk at M6 (*n* = 68)	55 (80.9%)		

*Data are median (25th−75th interquartile), or number (percentage). CIRS, Cumulative Illness Rating Scale; COPD, Chronic Obstructive Pulmonary Disease; CFS, Clinical Frailty Scale; ADL, Activity of Daily Living; IADL, Instrumental Activity of Daily Living*.

### Immunophenotyping and Function

#### Innate Compartment

**Figure 1:**
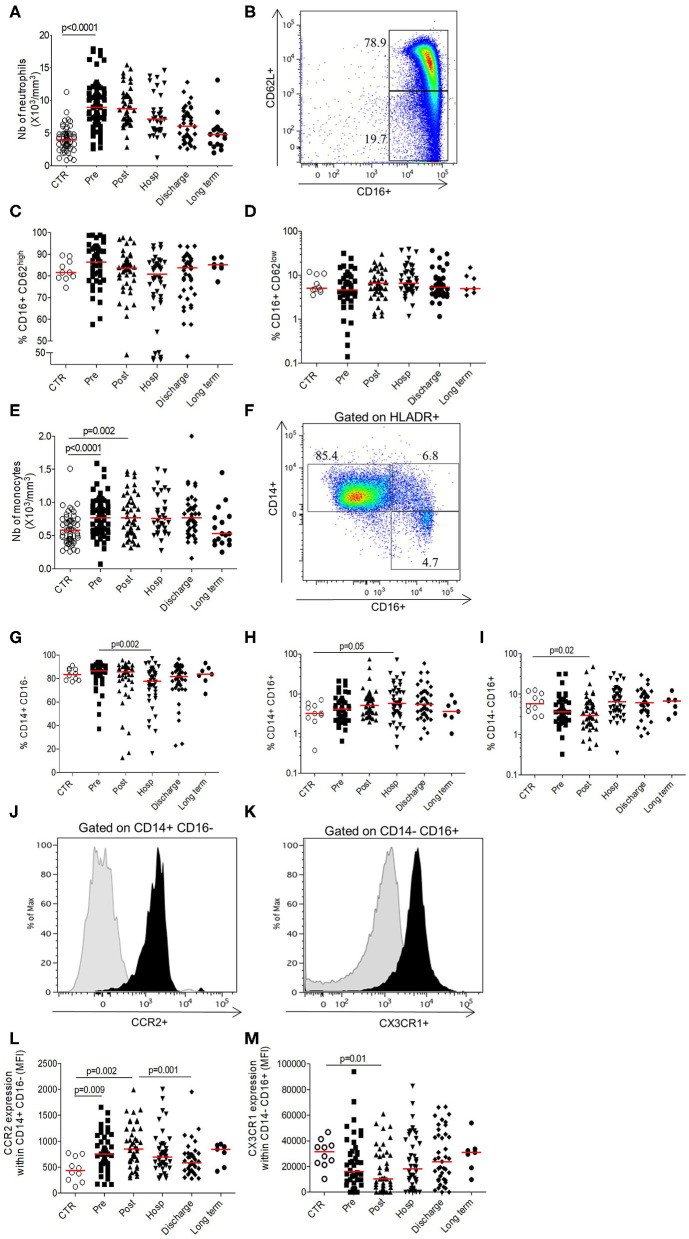
Longitudinal analysis of innate phenotype. **(A)** Number of neutrophils (10^3^ cells/mm^3^); **(B)** Gating strategy depicting neutrophils staining based on size/structure and CD62L/CD16 expression; **(C)** Percentage of inflammatory neutrophils expressing CD16^+^CD62L^high^; **(D)** Percentage of suppressive neutrophils expressing CD16^+^CD62L^low^; **(E)** Number of monocytes (10^3^cells/mm^3^); **(F)** Gating strategy depicting monocytes staining based on size/structure criteria, HLADR, CD14, and CD16 expression; **(G)** Percentage of conventional CD14^+^CD16^−^ monocytes; **(H)** Percentage of intermediate CD14^+^ CD16^+^ monocytes; **(I)** Percentage of non-conventional CD14^−^CD16^+^ monocytes; **(J)** Representative histogram of CCR2 expression within conventional monocytes (black; control isotype in gray overlay); **(K)** Representative histogram of CX3CR1 expression within non-conventional monocytes (black; control isotype in gray overlay); **(L)** CCR2 expression within conventional monocytes (expressed in mean fluorescence intensity); **(M)** CX3CR1 expression within non-conventional monocytes (expressed in mean fluorescence intensity). Data are plotted for age-matched control individuals (CTR) or for hip fracture patients at different times of follow-up (pre-surgery: PRE; post-surgery: POST; during hospitalization: HOSP; at hospital discharge: DISCHARGE and between 6 and 12 months post-fracture: LONG TERM). Each dot represents an individual. The lanes indicate the medians. Statistical significance is determined by the nonparametric Mann–Whitney test: *p* < 0.05 was considered significant.

The total neutrophils and monocytes counts transitory increase in the pre-operative period comparatively to controls (Neutrophils 8.9 × 10^6^/mm^3^ [7–10.9] vs. 3.9 × 10^6^/mm^3^ [3–4.9], *p* < 0.0001; Monocytes 0.77 × 10^6^/mm^3^ [0.57–0.94] vs. 0.58 × 10^6^/mm^3^ [0.43–0.72], *p* < 0.0001) before cell counts normalization and return to baseline at long term (Figures 1A,E), suggesting an immediate mobilization of the innate compartments.

Neutrophils subsets were gated on size and structure as well as the combination of CD16+ and CD62L (Figure 1B) in order to differentiate inflammatory neutrophils (CD16+CD62L^high^; Figure 1C) from anti-inflammatory neutrophils (CD16+CD62L^low^; Figure 1D). Their respective proportions were not significantly different from the control group (Figures 1C,D). Gating strategy of monocytes was represented in the Figure 1F and relies on the combination of size, structure, HLA-DR, CD14, and CD16 expression. The proportion of intermediary monocytes (CD14+CD16+), known as “inflammatory” monocytes (18), increased during hospitalization before return to baseline at long term (Figure 1H). On the contrary, the proportions of conventional and non-conventional monocytes decreased in post-operative period and during hospitalization comparatively to control group and pre-operative period respectively (Figures 1G,I). Concerning CCR2 and CX3CR1 chemokines, known to be differentially expressed according to cell subsets, we observed that the expression of CCR2 within conventional monocytes increased significantly after HF to be maximal in the post-operative period (MFI 849 [637–1,211] vs. 442 [248–728], *p* = 0.002) (Figures 1J,L) and this trend is the same whatever the monocytes subset (Supplementary Figures 1A,B). Inversely, the expression of CX3CR1 transitory decreased after HF to be minimal at the post-operative timepoint (MFI 10400 [3,120–28,896] vs. 31,427 [22,395–37,612], *p* = 0.01; Figures 1K,M) with the same trends for the other monocytes subset (Supplementary Figures 1C,D). These results suggest an increased turnover of monocyte/macrophage precursors in the bone marrow (decrease of CX3CR1 expression) (19) and an elevated monocyte migration from bone marrow to inflammatory site (increase of CCR2 expression).

**Figure 2:**
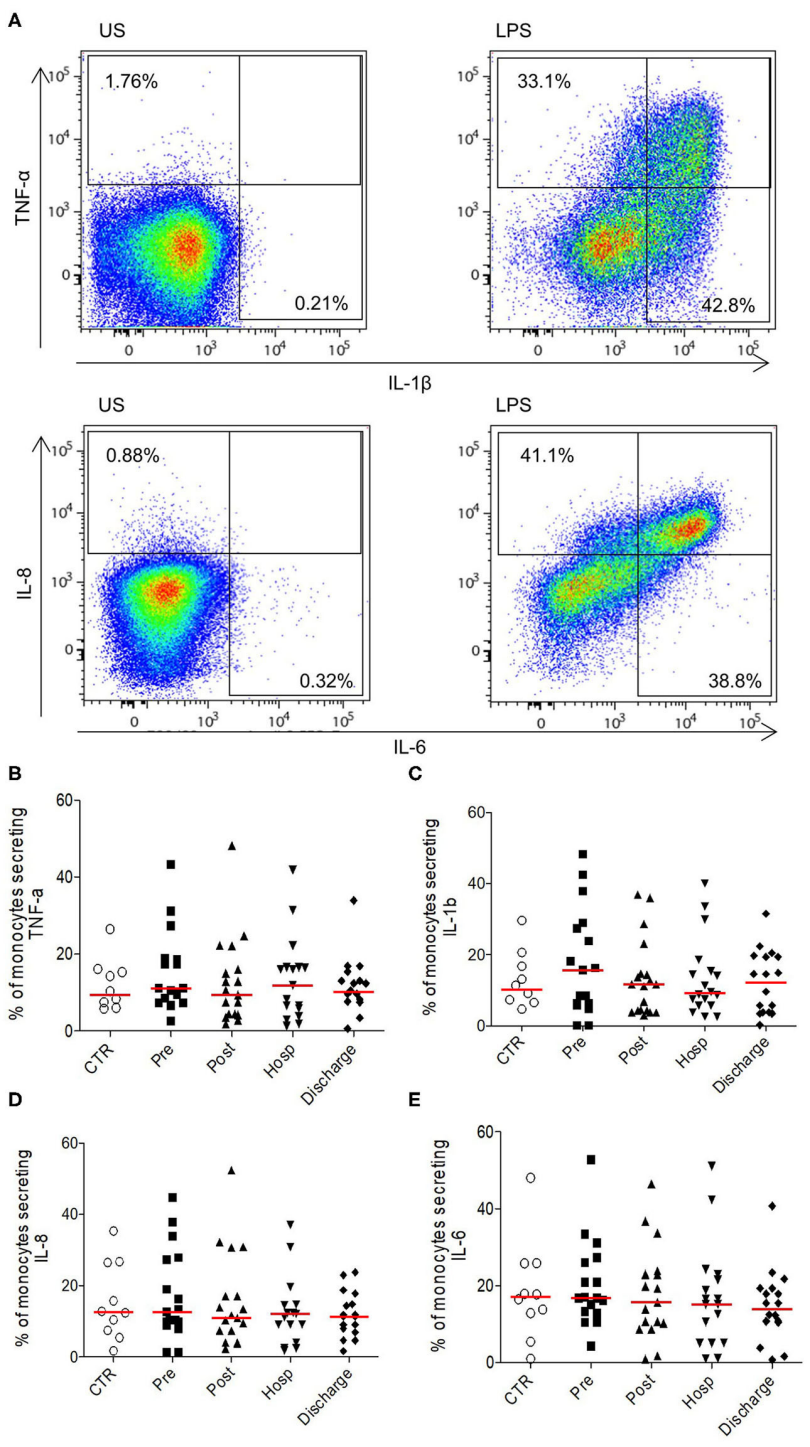
Ability of monocytes from HFP to produce pro-inflammatory cytokines upon stimulation. **(A)** Representative flow cytometry profile of cytokines secretion (TNF-α and IL-1β upper panel; IL-8 and IL-6 bottom panel) in unstimulated monocytes (US, left panel) or in LPS conditions (right panel). Monocytes were identified according to size/structure, HLADR, CD14, and CD16 expression; **(B)** Percentages of monocytes secreting **(B)** TNF-α, **(C)** IL-1 β, **(D)** IL-8, and **(E)** IL-6. Data are plotted for age-matched control individuals (CTR) or for hip fracture patients at different times of follow-up (pre-surgery: PRE; post-surgery: POST; during hospitalization: HOSP; at hospital discharge: DISCHARGE and between 6 and 12 months post-fracture: LONG TERM). Each dot represents an individual. The lanes indicate the medians. Statistical significance is determined by the nonparametric Mann–Whitney test: *p* < 0.05 was considered significant.

Overall, these results show a transitory activation of the innate compartment after HF, followed by a normalization of the different phenomenons leading to a homeostatic return at long term. To evaluate monocytes function after HF, we analyzed their pro-inflammatory cytokines production under LPS stimulation. At each time points, monocytes were able to secrete IL-1β, IL-6, IL-8, and TNF-α without significant difference compared to age-matched controls (Figure 2). Therefore, despite the hyper-inflammatory context of acute HF, functionality of monocytes to induce pro-inflammatory signals in response to acute stress is preserved after HF.

#### Adaptive Compartment

**Figure 3:**
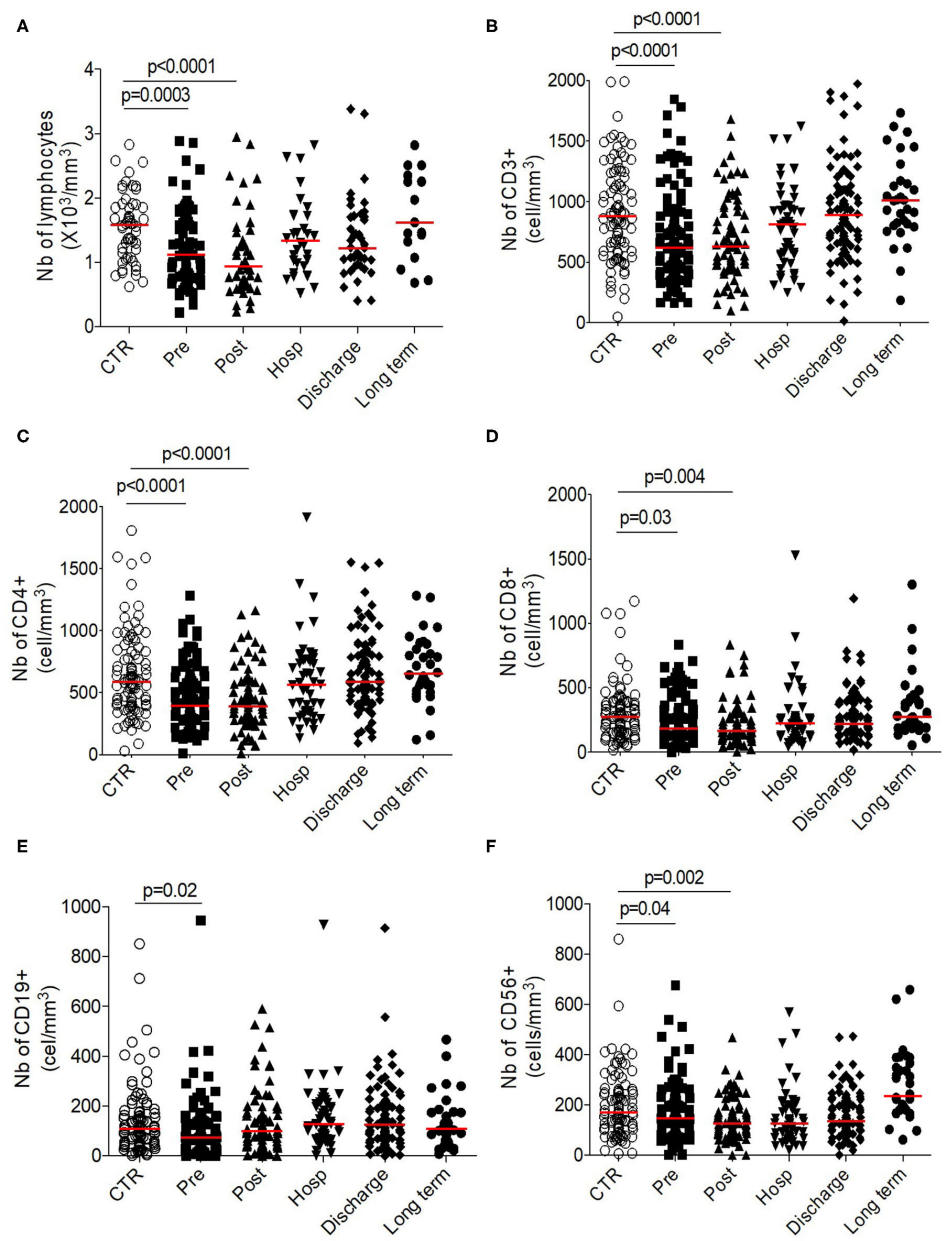
Longitudinal analysis of adaptive phenotype. Number (10^3^ cells/mm^3^) of **(A)** total lymphocytes, **(B)** T-cells, **(C)** CD4+ T-cells, **(D)** CD8+ T-cells, **(E)** CD19+ B cells, and of **(F)** CD56+ NK cells. Data are plotted for age-matched control individuals (CTR) or for hip fracture patients at different times of follow-up (pre-surgery: PRE; post-surgery: POST; during hospitalization: HOSP; at hospital discharge: DISCHARGE and between 6 and 12 months post-fracture: LONG TERM). Each dot represents an individual. The lanes indicate the medians. Statistical significance is determined by the nonparametric Mann–Whitney test: *p* < 0.05 was considered significant.

Contrary to the innate cell subsets, we observed a significant and transitory lymphopenia after HF compared to control group (post-operative 0.94 × 10^6^/mm^3^ [0.66–1.37] vs. control 1.59 × 10^6^/mm^3^ [1.13–1.9], *p* < 0.0001; Figure 3A). This lymphopenia is significantly observed on T cells (CD3+, both for CD4+ and CD8+ cells; Figures 3B–D), on B cells (CD19+) (Figure 3E) and on NK cells (CD3–CD56+) (Figure 3F). Of note, all subsets seem to be differentially affected: the lymphopenia is more sustained for the CD4+ proportionally to the CD8+ compartment; B cells recover faster during hospitalization whereas NK cells are mobilized at early timepoints post-fracture (Supplementary Figure 2).

Altogether, these results suggest a transitory lymphosuppressive profile within adaptive compartment after HF.

**Figure 4:**
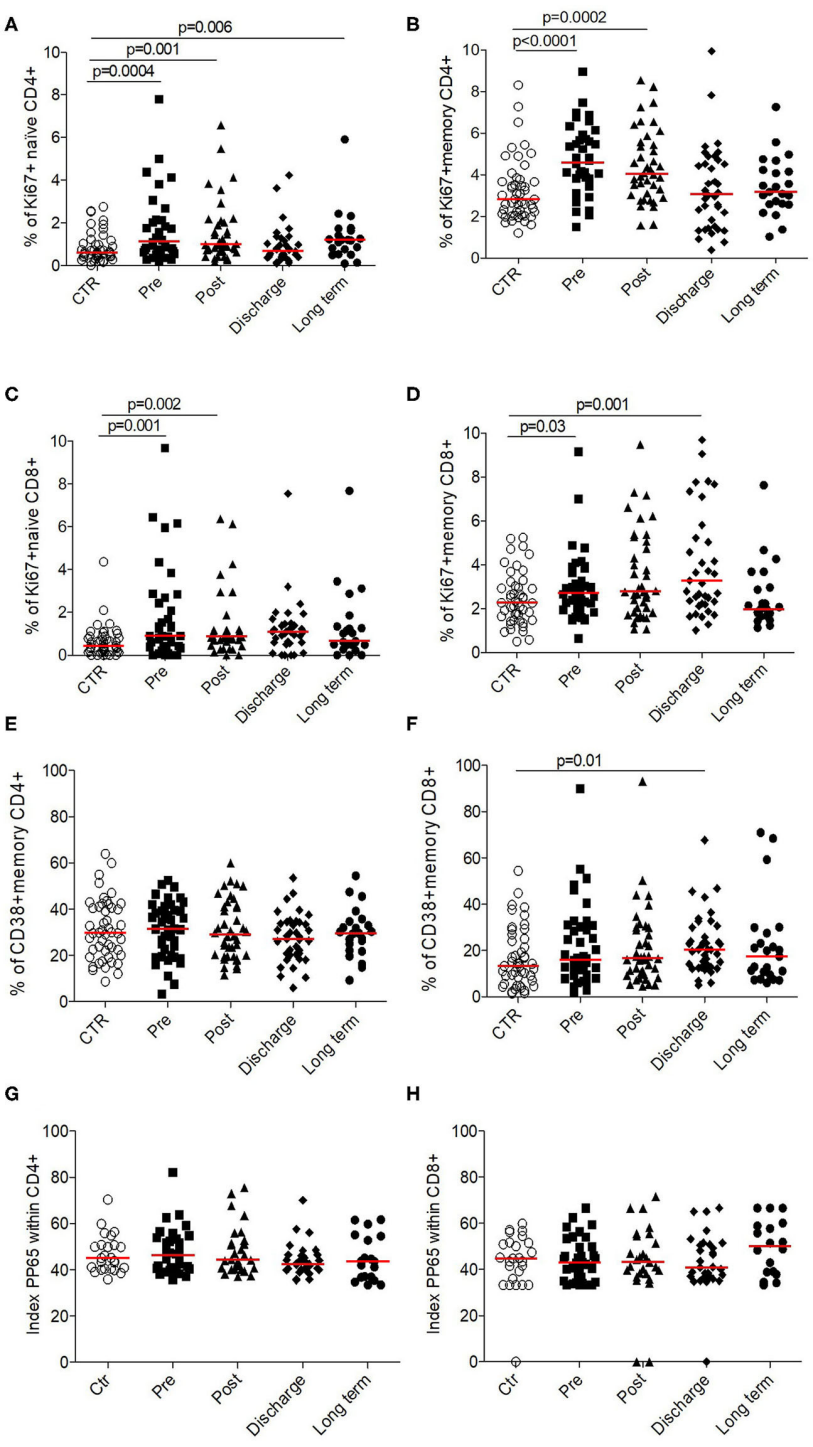
Functionality of CD4^+^ and CD8^+^ T cells in HF patients (longitudinal analysis) compared to control. Proliferation was evaluated by the % of cells expressed Ki67^+^ for **(A)** CCR7+ CD45RA+ naïve CD4^+^, **(B)** memory CD4^+^, **(C)** CCR7+ CD45RA+ naïve CD8^+^, and **(D)** memory CD8^+^. Activation was evaluated by the % of cells expressed CD38+ within **(E)** memory CD4^+^ and **(F)** CD8^+^. Priming capacity was evaluated by the polyfunctionality index within **(G)** CD4^+^ and **(H)** CD8^+^. Data are plotted for age-matched control individuals (CTR) or for hip fracture patients at different times of follow-up (pre-surgery: PRE; post-surgery: POST; during hospitalization: HOSP; at hospital discharge: DISCHARGE and between 6 and 12 months post-fracture: LONG TERM). Each dot represents an individual. The lanes indicate the medians. Statistical significance is determined by the nonparametric Mann–Whitney test: *p* < 0.05 was considered significant.

To evaluate if this lymphopenic state led to compensatory mechanisms such as lymphopenia-induced proliferation through homeostatic signals, we measured their *ex vivo* proliferation capacity (Ki67 level) on naïve and memory T cell compartments. Whatever the subsets followed, we observed that T-cells isolated from elderly patients suffering from HF were proliferated during their stay to hospital in order to counteract the existing and persistent lymphopenia (Figures 4A–D).

This response to homeostatic signals was confirmed by the fact that T-cells were not activated (based on CD38+ expression; Figures 4E,F).

Despite the absence of CMV reactivation during this acute clinical event (data not shown), we decided to evaluate *in vitro* the functionality of lymphocytes by analyzing their ability to response to pp65 antigens (which constitute the immunodominant responses described for CMV infection in elderly). Thus, taking into account the proportion of either CD4+, either CD8+ T-cells to secrete IFN-γ and/or TNF-α and/or IL-2, we found that T-cells in HF patients were as polyfunctional as elderly controls (Figures 4G,H), suggesting that T-cells were fully functional and able to response to antigenic stimulation if necessary.

#### Immune Checkpoint Analysis

**Figure 5:**
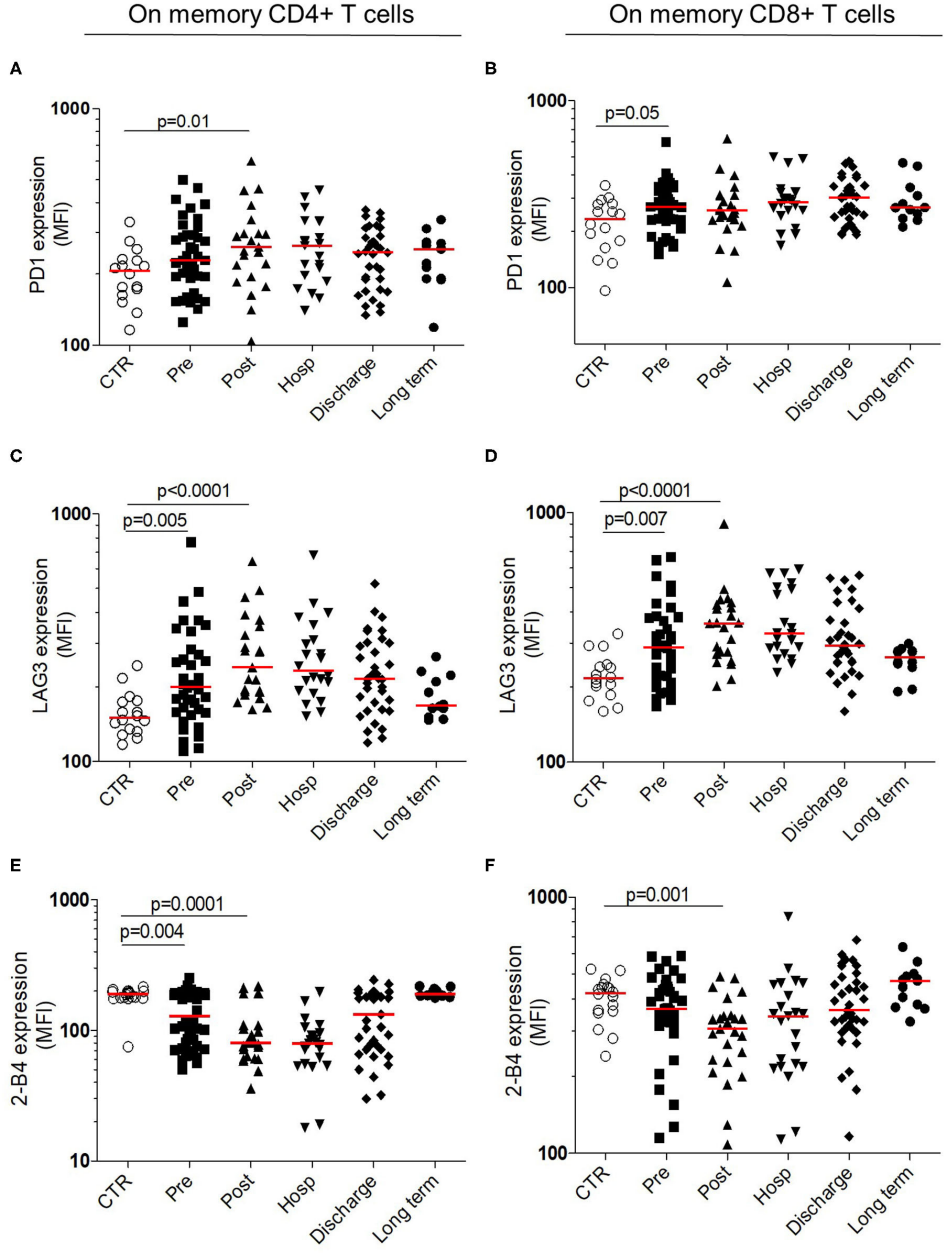
Longitudinal analysis of immune checkpoint expression within T cells. Surface membrane expression within memory CD4^+^ **(Left)** and memory CD8+ T cells **(Right)** for PD-1 (**A,B** upper panels) LAG-3 (**C,D** middle panels) and 2-B4 expression (**E,F** bottom panels). All results are expressed in mean fluorescence intensity. Data are plotted for age-matched control individuals (CTR) or for hip fracture patients at different times of follow-up (pre-surgery: PRE; post-surgery: POST; during hospitalization: HOSP; at hospital discharge: DISCHARGE and between 6 and 12 months post-fracture: LONG TERM). Each dot represents an individual. The lanes indicate the medians. Statistical significance is determined by the nonparametric Mann–Whitney test: *p* < 0.05 was considered significant.

Previous results suggest an immunosuppressive profile within the adaptive compartment, without defect in functional capacities of T cells. We hypothesized that T-cells regulation, mediated by immune checkpoints, could be defective. The membrane expression of immune checkpoints regulators (PD-1 and LAG-3 and 2-B4) were analyzed on memory CD4+ and CD8+ T cells (Figure 5). The expression of immune checkpoints inhibitors significantly increased after HF compared to control. At long term, their expression returned to controls level (Figures 5A–D). The maximal expression of LAG-3 was observed in post-operative timepoint within CD4+ (post-operative MFI: 240 [191–352] vs. controls MFI: 150 [133–176], *p* < 0.0001) and CD8+ (post-operative MFI: 360 [272–429] vs. controls MFI: 217 [190–245], *p* < 0.0001) T cells (Figures 5C,D). Conversely, the expression of 2-B4 significantly decreased after HF compared to controls, within CD4+ and CD8+ with a minimal expression at post-operative timepoint (post-operative MFI: 81 [62–107] vs. controls MFI: 190 [178–200], *p* = 0.0001 and post-operative MFI: 307 [230–345] vs. controls MFI: 423 [358–448], *p* = 0.001, respectively; Figures 5E,F).

These results reinforce the transitory suppressive status in adaptive compartment, occurring early after the HF and the recovery to the homeostatic status at long term.

## Discussion

The objective of this study was to pinpoint immune modifications occurring after HF which represents an acute assault that can accelerate abruptly the progressive health decline associated with aging.

In fact, HF constitutes an important geriatric problem with high level of mortality and loss of functional autonomy. Despite improvement of clinical care through specific orthopedic geriatric unit creation (20), part of mechanisms implicated in the bad prognosis of HF keep unknown and could be a key to enhance medical care.

Longitudinal analysis of immune modifications is a strength in our study. Indeed, the four early time points permitted to highlight that immune changes appear in 1st hours following HF. Most of the time, these alterations falling into place at hospital discharge or at long term. Another strength of this work is the size of the cohort and the population characteristics. Patients included are typical of geriatric population with age over 80 years old, multiple comorbidities, polymedication, and frailty.

Our main results are: an increased number of innate compartments (neutrophils, monocytes) with a higher proportion of inflammatory monocytes in the first days following HF; a transitory decreased number of NK cells, T, and B lymphocytes. Despite these differential mobilizations, cells keep their ability to respond to environmental stimuli or homeostatic signals.

Moreover, there is a transient alteration in the regulation of T-cells activation with an increase expression of immune checkpoint inhibitors and a decrease expression of immune checkpoint activators, as previously described in trauma by Laudanski et al. (21) and in sepsis by Zhang et al. (22).

Overall, there is shift to a pro-inflammatory phenotype in innate compartment and to an anti-inflammatory phenotype in the adaptive compartment in the first days after HF. All these modifications are transitory forming a regulation loop before return at homeostatic status at long term.

HF represents an intense acute stress in old patients. It induces systemic reaction and notably immune responses. Another model of acute stress in geriatric population is sepsis. For several years, immune modifications by sepsis are described, associating an intense pro-inflammatory phase called “cytokine storm” and a suppressive phase with some similarities with our results (22).

During sepsis, phenotypic changes in monocytes are quite similar to what we observed in HF where the increased proportion of CD16+ monocytes is associated with an decreased expression of CX3CR1 (23–25). In our study, we observed a decreased expression of CX3CR1 and an increased expression of CCR2 indicating an intense turnover and recruitment of monocytes from bone marrow, potentially enabling migration to the fracture site.

In the 1st days of sepsis, there is a major leukocytosis similar to what we observed in this study of HF. However, in sepsis, neutrophils acquire a pro-inflammatory profile associating a decrease in the expression of CD16 and L-selectin (26). We did not observe differences in the expression of CD16 and L-selectin in our cohort. One hypothesis could be the bacterial origin of sepsis that mobilized intensively neutrophils as first line of defense against bacteria.

Concerning the adaptive compartment, early stage of sepsis is associated with global lymphopenia (27). CD4+, CD8+, B cells, and NK cells drastically decrease. Furthermore, inhibitory immune checkpoint (PD-1 and LAG-3) expression within T cells increases leading to T cells impairment and inhibition of innate cell function (28). We observed similar results in our study reinforcing the hypothesis of immunosuppression within adaptive compartment. Thus, negative signaling could contribute to T-cell anergy in trauma patients, as suggested by Bandyopadhyay et al. (29). Similarly, PD-L1 blockade has been shown to improve immune dysfunction of spleen dendritic cells and T-cells in multiple organs dysfunction syndromes (30).

If immune scar observed in HF is similar to the one observed in sepsis, it could be interesting to consider these two common complications in elderly populations as unique models to propose strategies to restore immunity after prolonged stress-induced immune suppression. Most recently, the concept of metabolic dysfunction has emerged as a factor underlying impaired function of the innate and adaptive immune systems of severely injured patients (31).

A comprehensive assessment of immune mechanisms implicated in the patients prognosis after HF appears important and could pave the way to news immune therapeutics approach.

In this regard, a recent study elegantly showed that hip fracture and surgical trauma cause significant increases in PD-1 expression in aged mice compared to sham controls. Antibody blockade of PD-1 partially reverses T cell apoptosis, decreases the systemic inflammatory response and susceptibility to bacterial lung infection, and reduces mortality (32).

During sepsis, many approaches have been deployed to target immune checkpoints reviewed in (28) and represent therefore a nice model to understand underlying mechanisms improving clinical patients outcome. Targeting immune checkpoints which could potentially reverse innate and adaptive system hypo-responsiveness during the 1st days following hip fracture could benefit elderly patients in preventing and treating immune tolerance. However, such a therapy needs to be evaluated in this particular population, where advanced age of the individuals may play a role in their capacity to respond to treatment.

## Data Availability Statement

The raw data supporting the conclusions of this article will be made available by the authors, without undue reservation.

## Ethics Statement

The studies involving human participants were reviewed and approved by Comité de Protection des personnes (CPP) Pitié Salpêtrière, Paris, France. The patients/participants provided their written informed consent to participate in this study.

## Author Contributions

HV performed experiments, analyzed the data, and wrote the paper. CB, SF, and TF performed experiments and analyzed the data. HL, FK, and JB recruited patients and analyzed clinical data. JB designed clinical study, recruited patients, and analyzed clinical data. DS designed research, performed experiments, analyzed the data, and wrote the paper. All authors contributed to the article and approved the submitted version.

## Conflict of Interest

The authors declare that the research was conducted in the absence of any commercial or financial relationships that could be construed as a potential conflict of interest.

## Footnotes

^1^Available online at: https://www.dal.ca/sites/gmr/our-tools/clinical-frailty-scale.html.

## Supplementary Material

Supplementary Figure 1 Longitudinal analysis of chemokines expression within monocytes subtype. CCR2 expression within **(A)** intermediate and **(B)** non-conventional monocytes (expressed in mean fluorescence intensity). CX3CR1 expression within intermediate **(C)** and conventional **(D)** monocytes (result expressed in mean fluorescence intensity). Data are plotted for age-matched control individuals (CTR) or for hip fracture patients at different times of follow-up (pre-surgery: PRE; post-surgery: POST; during hospitalization: HOSP; at hospital discharge: DISCHARGE and between 6 and 12 months post-fracture: LONG TERM). Each dot represents an individual. The lanes indicate the medians. Statistical significance is determined by the nonparametric Mann–Whitney test: *p* < 0.05 was considered significant.

Supplementary Figure 2 Longitudinal analysis of adaptive phenotype. **(A)** % of CD4^+^ T cells, **(B)** % of CD8^+^ T cells, **(C)** % of CD19^+^, and **(D)** % of CD56^+^. Data are plotted for age-matched control individuals (CTR) or for hip fracture patients at different times of follow-up (pre-surgery: PRE; post-surgery: POST; during hospitalization: HOSP; at hospital discharge: DISCHARGE and between 6 and 12 months post-fracture: LONG TERM). Each dot represents an individual. The lanes indicate the medians. Statistical significance is determined by the nonparametric Mann–Whitney test: *p* < 0.05 was considered significant.
