# Supplementary figures and images for: Hip Fracture Leads to Transitory Immune Imprint in Older Patients
Source: Front Immunol. 2020 Sep 18;11:571759. doi: 10.3389/fimmu.2020.571759 (PMC7533556; doi:10.3389/fimmu.2020.571759)

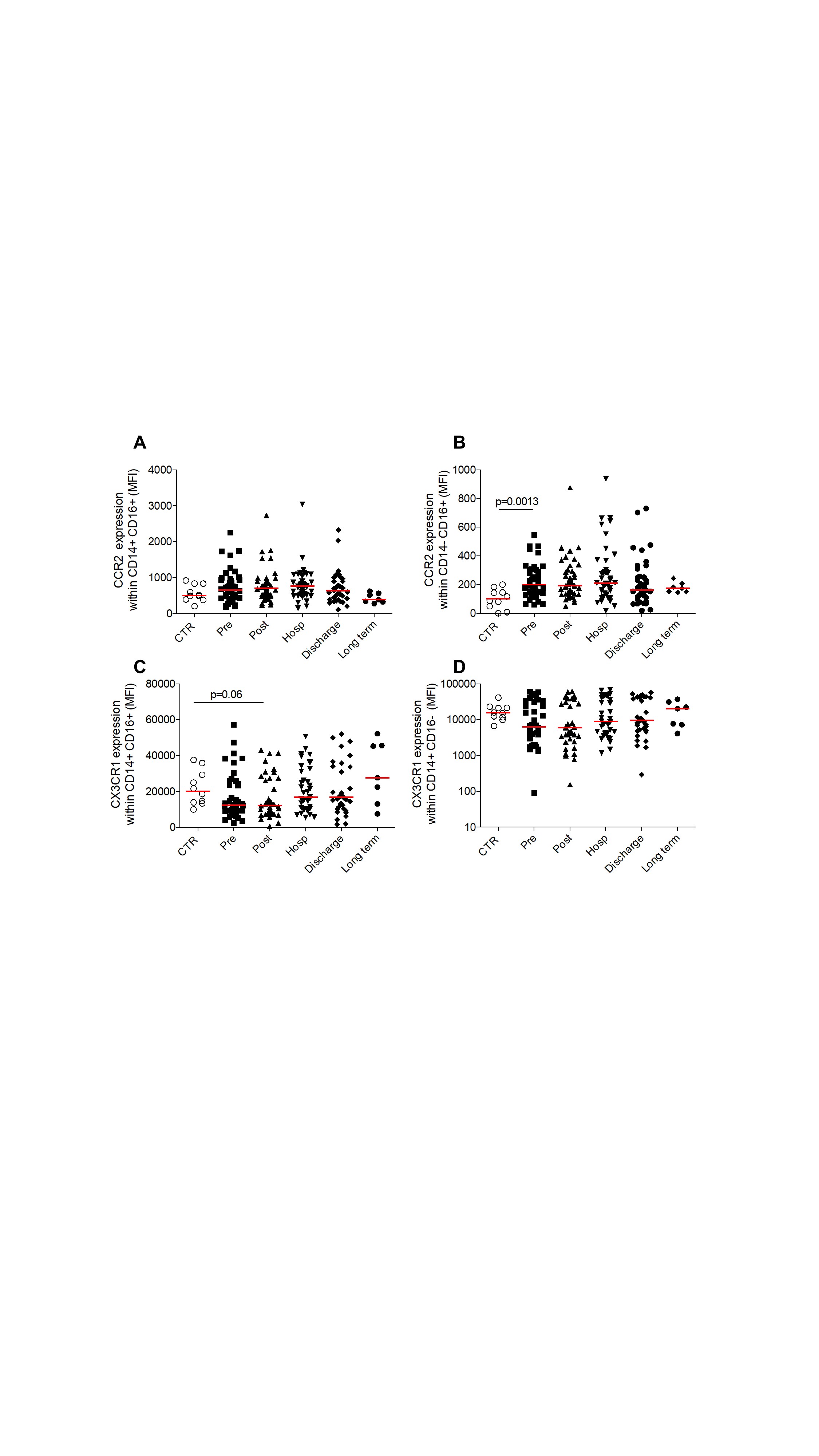

Supplement: Supplementary Figure 1 — Longitudinal analysis of chemokines expression within monocytes subtype. CCR2 expression within (A) intermediate and (B) non-conventional monocytes (expressed in mean fluorescence intensity). CX3CR1 expression within intermediate (C) and conventional (D) monocytes (result expressed in mean fluorescence intensity). Data are plotted for age-matched control individuals (CTR) or for hip fracture patients at different times of follow-up (pre-surgery: PRE; post-surgery: POST; during hospitalization: HOSP; at hospital discharge: DISCHARGE and between 6 and 12 months post-fracture: LONG TERM). Each dot represents an individual. The lanes indicate the medians. Statistical significance is determined by the nonparametric Mann–Whitney test: p < 0.05 was considered significant. [file Image_1.JPEG]

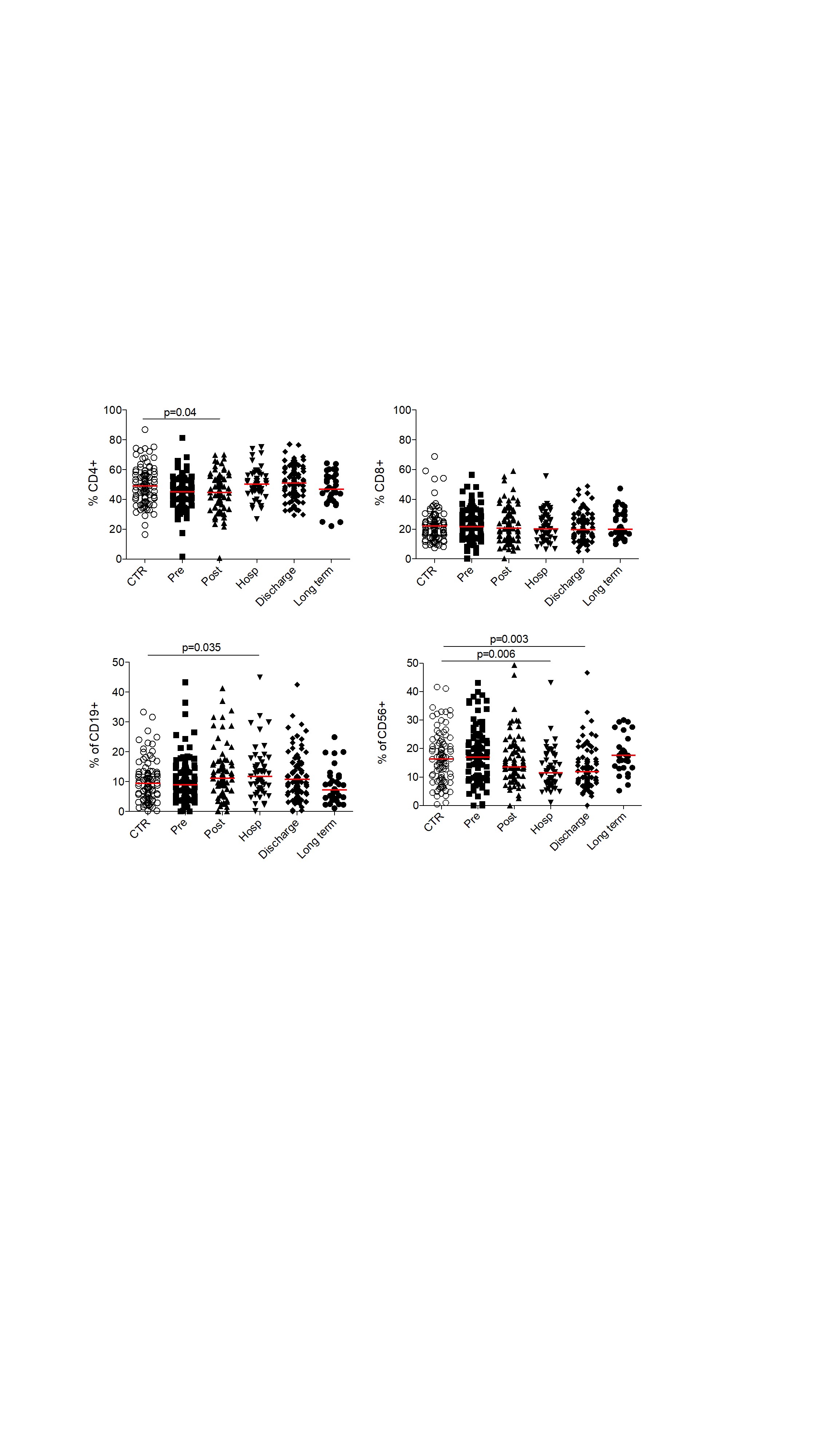

Supplement: Supplementary Figure 2 — Longitudinal analysis of adaptive phenotype. (A) % of CD4+ T cells, (B) % of CD8+ T cells, (C) % of CD19+, and (D) % of CD56+. Data are plotted for age-matched control individuals (CTR) or for hip fracture patients at different times of follow-up (pre-surgery: PRE; post-surgery: POST; during hospitalization: HOSP; at hospital discharge: DISCHARGE and between 6 and 12 months post-fracture: LONG TERM). Each dot represents an individual. The lanes indicate the medians. Statistical significance is determined by the nonparametric Mann–Whitney test: p < 0.05 was considered significant. [file Image_2.JPEG]
